# Role of MicroRNAs in the Pathogenesis of Coronary Artery Disease

**DOI:** 10.3389/fcvm.2021.632392

**Published:** 2021-04-12

**Authors:** Soudeh Ghafouri-Fard, Mahdi Gholipour, Mohammad Taheri

**Affiliations:** ^1^Department of Medical Genetics, Shahid Beheshti University of Medical Sciences, Tehran, Iran; ^2^Urology and Nephrology Research Center, Shahid Beheshti University of Medical Sciences, Tehran, Iran

**Keywords:** coronary artery disease, miRNA, expression, biomarkers, myocardial infarction

## Abstract

Coronary artery disease (CAD) is the main reason of cardiovascular mortalities worldwide. This condition is resulted from atherosclerotic occlusion of coronary arteries. MicroRNAs (miRNAs) are implicated in the regulation of proliferation and apoptosis of endothelial cells, induction of immune responses and different stages of plaque formation. Up-regulation of miR-92a-3p, miR-206, miR-216a, miR-574-5p, miR-23a, miR-499, miR-451, miR-21, miR-146a, and a number of other miRNAs has been reported in CAD patients. In contrast, miR-20, miR-107, miR-330, miR-383-3p, miR-939, miR-4306, miR-181a-5p, miR-218, miR-376a-3p, and miR-3614 are among down-regulated miRNAs in CAD. Differential expression of miRNAs in CAD patients has been exploited to design diagnostic or prognostic panels for evaluation of CAD patients. We appraise the recent knowledge about the role of miRNAs in the development of diverse clinical subtypes of CAD.

## Introduction

Coronary artery disease (CAD) is the principal source of cardiovascular mortalities worldwide (1). In 2020, it is expected that 11.1 million patients die as a results of CAD related complications (2). Clinically, CAD has different categories ranging from stable angina pectoris to acute coronary syndromes which comprises unstable angina (UA) and myocardial infarction (MI) (3). The majority of MI cases are resulted from the establishment of acute intraluminal coronary thrombus inside an epicardial coronary artery and the subsequent occlusion of the coronary artery (4, 5). The acute coronary thrombosis results in a sudden decrease in the blood flow and induction of necrosis in the myocardial region which is takes the blood supply from this coronary artery (6). Some other cardiovascular pathologies might be associated with CAD. For instance, acute MI might lead to defects in functioning myocytes resulting in myocardial fibrosis and left ventricle dilatation. Subsequent induction of neurohormonal responses and left ventricle remodeling results in progressive weakening of the residual viable myocardium (7). Moreover, ischemic conditions leads to upsurge of endogenous catecholamines in the myocardial interstitial fluid which in turn increases myocardial apoptosis and fibrosis (8). Dysregulation of several microRNAs (miRNAs) has been displayed in different categories of CAD, potentiating these transcripts as biomarkers of this devastating condition (9). miRNAs have been shown to modulate gene expression at post transcriptional level via destroying mRNA targets or by obstructing their translation (10). Since each miRNA is capable of regulating expression of several transcripts, it is estimated that more than half of protein-coding genes in the human genome are influenced by miRNAs (11). Therefore, miRNAs can affect numerous important biological and cellular function such as cell differentiation, proliferation, and cell death in the cardiovascular system (12). Understanding the role of miRNAs in the pathogenesis of CAD would lead to identification of appropriate therapies for this global health problem. We appraise the recent knowledge about the role of miRNAs in the development of diverse clinical subtypes of CAD.

## miRNAs in CAD

**Figure 1:**
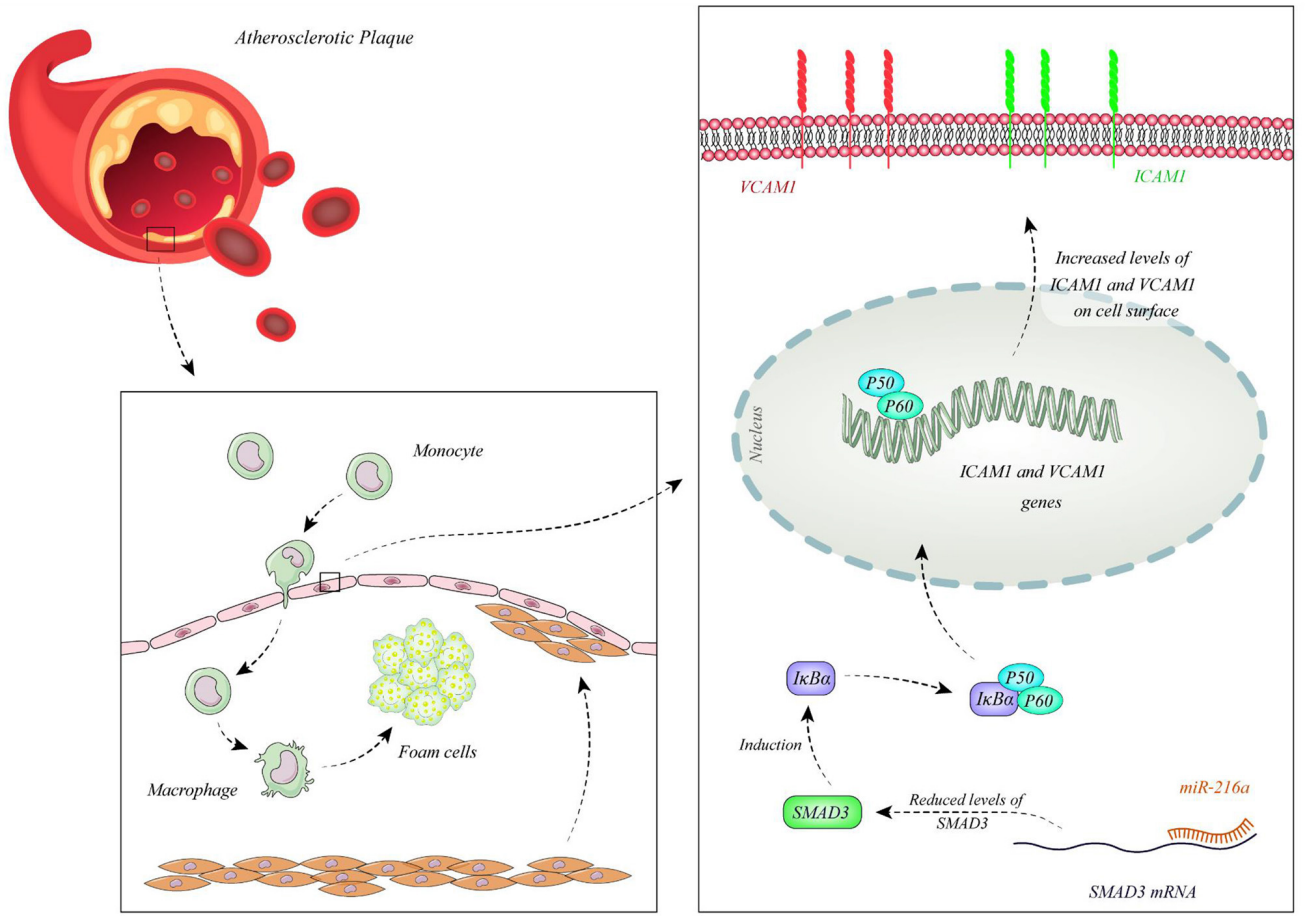
miR-216a is over-expressed in CAD patients. miR-216a attaches to 3' UTR of Smad3 and decreases its levels. Down-regulation of Smad3 leads to reduction of IκBα releasing NF-κB and enhancing its nuclear transport. Subsequent up-regulation of ICAM1 and VCAM1 enhances attachment of monocytes to endothelial cells promoting development of CAD (16).

Function of miRNAs in CAD has been assessed in different cell types. Endothelial cells have been the mostly assessed cell type in this regard. Liu et al. have extracted circulating microvesicles (MVs) from plasma samples of CAD patients to assess signature of their miRNA constituents. Among miRNAs which were reported to regulate vascular performance, miR-92a-3p has been shown to be up-regulated in CAD cases compared with non-CAD individuals. MVs enclosing miR-92a-3p have been demonstrated to be mostly originated from endothelial cells. Treatment of these cells with oxidized LDL and IL-6 has resulted in up-regulation of miR-92a-3p levels in these cells and higher incorporation of this miRNA in MVs. Transport of these MVs to other endothelial cells has enhanced their migration and proliferation. miR-92a-3p exerts these functions through inhibition of expression of THBS1, the inhibitor of angiogenesis. Taken together, atherosclerosis enhances the incorporation of endothelial miR-92a-3p into MVs, which controls angiogenesis in recipient endothelial cells through a THBS1-associated route (13). Wang et al. have demonstrated up-regulation of miR-206 in endothelial progenitor cells as well as plasma samples gathered from CAD patients. However, expression levels of miR-206 have not been associated with clinicopathological characteristics of CAD patients. Functionally, miR-206 has been shown to inhibit the viability and invasion of endothelial progenitor cells in CAD patients, while enhancing apoptosis in these cells. miR-206 can also suppress expression of vascular endothelial growth factor (VEGF) (14). Moreover, this miRNA modulates endothelial progenitor cell functions through targeting the protein kinase PIK3C2α. This protein kinase has been shown to be down-regulated in endothelial progenitor cells of CAD patients. miR-206 silencing in these cells enhanced their angiogenic and vasculogenic capacities both *in vitro* and in an animal model of ischemia. Besides, miR-206 silencing enhanced activities of PIK3C2α, Akt, and endothelial nitric oxide synthase (15). miR-216a is another miRNA which is involved in endothelial aging and dysfunction through modulating expression of Smad3. Over-expression of miR-216a in human umbilical vein endothelial cells (HUVECs) has activated an untimely senescence-like feature in these cells which was accompanied by defects in proliferation and migration. The consequent suppression of Smad3 has resulted in enhancement of adhesion of these cells to monocytes, modulation of the destruction of NF-κB inhibitor alpha (IκBα) and stimulation of adhesion proteins. Levels of miR-216a has been shown to be elevated in the plasma samples of old CAD patients in association with higher susceptibility to CAD (16). Figure 1 shows the cascade of involvement of miR-216a in CAD.

Gao et al. have demonstrated high concentrations of lipids, atherosclerotic index, apoptotic index, and KRT1-positive expression while suppression of Notch signaling pathway in the atherosclerotic mice. miR-107 has been shown to bind with KRT1, thus reducing its expression. This miRNA has been down-regulated in animal models of CAD (17). Ren et al. have reported down-regulation of miR-330 in CAD group. Overexpression of miR-330 has been shown to inhibit atherosclerotic plaques creation whereas enhancing proliferation of vascular endothelial cells through modulating MAPK8 via the WNT signaling pathway (18). Lian et al. have shown down-regulation of miR-383-3p and up-regulation of IL1R2 in myocardial tissues of atherosclerotic animals. Forced over-expression of miR-383-3p has reduced expression of IL1R2, caspase-1, IL-1β, IL-6, and IL-18, ameliorated cell apoptosis in the coronary artery endothelial cells, while enhanced IL-10 levels, cell survival, and tube construction (19). Hou et al. have reported down-regulation of miR-939 in the blood of patients with adequate coronary collateral circulation compared with those having insufficient coronary collateral circulation. Up-regulation of miR-939 in HUVECs has remarkably suppressed proliferation, adhesion and tube construction, while increasing migration capacity of these cells. γ-catenin has been identified as a direct target of miR-939 (20). Expressions of both miR-181a-5p and miR-181a-3p have been lower in the aorta plaque and plasma of animal models of CAD. Up-regulation of these miRNAs considerably delays atherosclerotic plaque development in animals. These miRNAs have functional roles in the reduction of expression of pro-inflammatory proteins and diminishing the infiltration of macrophage, leukocyte and T cell into the atherosclerotic plaques through suppression of adhesion molecule expressions in HUVECs (21). miR-376a-3p has also been down-regulated in CAD samples. *In vitro* studies have shown the effects of miR-376a-3p silencing in the suppression of proliferation of HUVECs through modulating NRIP1 expression (22). Table 1 displays the functional roles of miRNAs in the development of CAD, based on the results of studies which have been conducted in endothelial cells.

**Table 1:** CAD-related miRNAs whose function has been assessed in endothelial cells.

**microRNA**	**Samples**	**Expression pattern**	**Assessed cell lines**	**Gene/protein interactions**	**Signaling pathway**	**Function**	**References**
*miR-92a-3p*	Plasma circulating microvesicles from 41 angiographically excluded CAD patients, 77 patients with stable CAD and 62 patients with acute coronary syndrome	Up-regulated	ECs	THBS1	–	Its knockdown attenuates migration and proliferation of endothelial cells through increasing THBS1 expression	(13)
*miR-206*	Blood samples from 78 patients with CAD and 65 healthy controls	Up-regulated	EPCs (endothelial progenitor cells)	VEGF	–	Inhibits invasion and cell viability in EPCs can suppress expression of VEGF	(14)
*miR-206*	Endothelial progenitor cells collected from peripheral blood of 53 CAD patients and 34 healthy controls, Nude mice	Up-regulated	EPCs	PIK3C2α	–	Reduces migration and its knockdown rescued angiogenic and vasculogenic abilities of endothelial progenitor cells	(15)
*miR-216a*	Blood samples from 176 patients with CAD and 342 age-matched control individuals	Up-regulated	HUVECs	Smad3	–	Promotes monocytes adhesion, endothelial senescence and inflammation through regulating Smad3/IκBα axis	(16)
*miR-499*	Plasma samples from 216 CAD patients and 90 healthy individuals	Up-regulated	HUVECs	PDCD4	NF-Kβ/TNF-α signaling pathway	Promotes apoptosis rate and decreases survival rate of endothelial cells by reducing expression of PDCD4	(23)
*miR-451*	Blood samples form 30 patients with coronary heart disease and 30 healthy controls	Up-regulated	HUVECs	VEGFA	PI3K-Akt-mTOR pathway	Suppresses cell proliferation and induces apoptosis in HUVECs by targeting VEGFA	(24)
*miR-107*	80 specific-pathogen-free (SPF) Kunming mice	Down-regulated	vascular endothelial cells	KRT1	Notch signaling pathway	Its overexpression decreases apoptosis and inflammation so prevents atherosclerosis by targeting KRT1 and activating Notch signaling pathway	(17)
*miR-330*	Female specific pathogen free (SPF) rats with acute coronary syndrome	Down-regulated	vascular endothelial cells	MAPK8	WNT signaling pathway	Its overexpression inhibits formation of atherosclerotic plaques and promotes proliferation of vascular endothelial cells by targeting MAPK8	(18)
*miR-939*	Blood samples from 25 CAD patients with poor CCC and 22 CAD patients with sufficient CCC	Down-regulated	HUVECs	γ-catenin	–	Suppresses angiogenesis and abrogates vascular integrity by targeting γ-catenin	(20)
*miR-181a-5p* *miR-181a-3p*	Plasma samples from 15 CAD patients and 20 healthy controls, ApoE^−/−^ mice	Down-regulated	HUVECs	TAB2, NEMO	NF-κB signaling pathway	miR-181a-5p and miR-181a-3p overexpression prevents endothelium inflammation and atherosclerosis progression by targeting TAB2 and NEMO, respectively. Also they suppresses expression of adhesion molecule	(21)
*miR-376a-3p*	Analysis of gene and microRNA expression profile datasets	Down-regulated	HUVECs	NRIP1	–	Its overexpression augmented cell proliferation by targeting NRIP1 in NRIP1	(22)
*miR-495*	Plasma samples form 30 CAD patients and 30 age and sex matched healthy controls	Down-regulated	HUVECs	CCL2	–	Regulated apoptosis and proliferation of HUVECs by targeting CCL2	(25)
*miR-383-3p*	30 male Sprague-Dawley (SD) rats with coronary artery atherosclerosis	Down-regulated	Coronary artery endothelial cells	IL1R2	–	Its upregulation reduces inflammatory cytokines expression and apoptosis rate in homocysteine-induced coronary artery endothelial cells by interacting with IL1R2	(19)
*miR-218*	Serum samples from 104 CAD patients and 101 healthy controls	Down-regulated	cardiac microvascular endothelial cells	–	–	Its upregulation promotes angiogenesis, cell proliferation and migration, enhances apoptosis rate and decreases inflammatory injury to CMECs	(26)

Lai et al. have reported over-expression of miR-574-5p in the serum samples and vascular smooth muscle cells (VSMCs) of CAD patients. Up-regulation of miR-574-5p has enhanced cell proliferation and suppressed apoptotic processes in VSMCs through targeting ZDHHC14 (27). Down-regulation of miR-146a has been demonstrated to attenuate apoptosis of vascular smooth muscle cells. Autologous injection of endothelial stem cells in a rat model of acute myocardial infarction has led to downregulation of miR-146a levels, reduction of apoptosis in the myocardial cells and decrease in infarct area. Such effects have been accompanied by up-regulation of VEGF (28). Expression of miR-93 has been increased in ventricle tissues and blood samples of mice model of MI. Moreover, miR-93 has been shown to be released from cardiomyocytes cultured in hypoxic conditions. miR-93 suppresses apoptotic processes and guards cardiomyocytes from ischemia/reperfusion damage. miR-93 silencing has deteriorated cardiac remodeling in these animal models. Thus, miR-93 over-expression and release from cardiomyocytes has been regarded as an adaptive mechanism following MI to attenuate cardiac remodeling and heart failure (29). miR-448 has been shown to be over-expressed in vascular smooth muscle cells (VSMCs) obtained from atherosclerotic plaques of coronary artery compared with those obtained from normal arteries. Expression of this miRNA is induced by PDGF-bb, a growth factor that enhances proliferation of VSMCs. MEF2C has been recognized as a direct target of miR-448 in VSMCs, though its down-regulation miR-448 enhances VSMCs migration (30). Table 2 shows the list of CAD-related miRNAs whose function has been assessed in myocardial cells or vascular smooth muscle cells.

**Table 2:** CAD-related miRNAs whose function has been assessed in myocardial cells or vascular smooth muscle cells.

**microRNA**	**Samples**	**Expression pattern**	**Assessed cell lines**	**Gene/protein interactions**	**Signaling pathway**	**Function**	**References**
*miR-574-5p*	Serum samples from 32 CAD patients and 30 normal individuals	Up-regulated	VSMCs	ZDHHC14	–	Suppresses apoptosis and promotes cell proliferation in VSMCs through targeting ZDHHC14	(27)
*miR-146a*	20 female Sprague-Dawley rats	Up-regulated	Myocardium	–	–	Injection of endothelial stem cell to rats with acute myocardial infarction caused decreased miR-146a expression and decreased cardiac apoptosis	(28)
*miR-93*	male C57BL/6 mice established as myocardial infarction (MI) models	Up-regulated	Cardiomyocytes	–	–	Suppresses apoptosis and promotes angiogenesis. Also has antioxidant effects	(29)
*miR-448*	atherosclerosis plaques and normal coronary artery tissues	Up-regulated	VSMCs	MEF2C	–	Promotes migration and proliferation of VSMCs by targeting MEF2C	(30)

Wang et al. have reported down-regulation of miR-20 in animal models of CAD in association with over-expression of VEGF and PTEN. Levels of miR-20a have been up-regulated following exercise in CAD animals. Up-regulation of miR-20a has reduced levels of ET-1, TxA2, ANGII, PTEN and enhanced levels of eNOS, PGI2, and VEGF. miR-20a exerts its functions through binding with the 3′UTR of PTEN, thus enhancing cell survival and proliferation via induction of the PI3K/Akt signaling (31). Expression of miR-4306 has been decreased in platelets and platelet-originated microparticles of CAD patients. Plasma miRNA-4306 has been mostly fractionated with microparticles rather than Argonaute2 complexes or HDL. These microparticles have the ability to transfer miR-4306 into human monocyte-derived macrophages, thus suppressing their migration and decreasing the quantity of macrophages in cardiac tissue in mouse model of MI. Mechanistically, miR-4306 binds with VEGFA to suppress ERK/NF-κB signaling (32). Expression of miR-23a has been higher in the peripheral blood mononuclear cells (PBMCs) of CAD patients compared with control subjects parallel with down-regulation of TRF2 levels. Aggressive lipid lowering therapy has reduced miR-23a, enhanced TRF2 expression and attenuated telomere erosion through this route (33). Expression of miR-3614 has been decreased by lipopolysaccharide (LPS) in macrophages, while LPS-associated inflammatory damage can be attenuated by up-regulation of miR-3614. This miRNA has been shown to target TRAF6 and suppress phosphorylation of kinases in the MAPK and NF-κB cascades Therefore, miR-3614/TRAF6/MAPK/NF-κB cascade can suppress devastating inflammatory responses (34). Animal studies have shown the role of miR-16 in reduction of development of atherosclerotic plaques and suppression of accretion of inflammatory factors while enhancement of release of anti-inflammatory factors. Mechanistically, miR-16 exerts these effects through downregulation of PDCD4 and activation of p38 and ERK1/2, while inactivation of JNK pathway (35). Table 3 demonstrates the relevance of miRNAs with the pathogenesis of CAD through summarizing the results of studies which reported function of miRNAs in macrophages/monocytes.

**Table 3:** CAD-related miRNAs whose function has been assessed in macrophages/ monocytes.

**microRNA**	**Samples**	**Expression pattern**	**Assessed cell lines**	**Gene/protein interactions**	**Signaling pathway**	**Function**	**References**
*miR-23a*	Blood samples (PBMCs) from 104 CAD patients and 50 control subjects	Up-regulated	PBMCs	TRF2	–	Contributes to telomere shortening and cellular senescence through targeting TRF2	(33)
*miR-4306*	Blood samples (platelet-derived microparticles) form CAD patients (24 AMI patients and 16 patients with stable angina pectoris) and 20 controls, C57BL/6 mice	Down-regulated	Primary human monocyte-derived macrophages	–	VEGFA/ERK1/2/NF-κB signaling pathways	Suppresses migration of HMDMs by regulating VEGFA/ERK1/2/NF-κB signaling pathways	(32)
*miR-3614*	epicardial adipose tissue from 30 CAD patients and 30 controls	Down-regulated	THP-1 (monocyte)	TRAF6	–	Its overexpression regulated inflammatory responses by targeting TRAF6	(34)
*miR-124*	Plasma samples from 40 patients with CAD and 40 non-CAD individuals, ApoE^−/−^ C57B/L6J mice	Down-regulated	RAW264.7 (mouse macrophage cell line)	p38	MAPK signaling pathway	Its overexpression decreased expression of pro-inflammatory cytokines and enhanced expression of anti-inflammatory cytokines	(36)
*miR-16*	Blood samples (plasma and PBMCs) from 40 patients with CAD and 40 non-CAD patients, 22 ApoE^−/−^ mice	Down-regulated	Peripheral blood mononuclear cells	PDCD4	–	Its overexpression Suppresses atherosclerotic plaque formation and proinflammatory factors secretion and promotes release of anti-inflammatory factors	(35)
*miR-21*	Circulating monocytes from CAD patients and non-CAD patients, apoE^−/−^ mice and miR-21^−/−^apoE^−/−^ mice	Up-regulated	Bone-marrow-derived macrophage	Dusp-8	–	Its knockout in mice caused decreased atherosclerotic lesions and smooth muscle cells in aorta also reduced macrophage migration and macrophage-endothelium interaction.	(37)

## Diagnostic/Prognostic Significance of miRNAs in CAD

Altered levels of miRNA in the circulation of CAD patients potentiates their usage as biomarkers in this condition. Zhong et al. have demonstrated differential expressions of tens of miRNAs in patients with UA or ST-segment elevation MI compared with normal controls. Receiver operating characteristics (ROC) curves have revealed miR-142-3p and miR-17-5p as possible markers for diagnosis of these two classes of CAD. Moreover, differential expressed miRNAs have been correlated with the pathological events during the course of CAD (38). Vahed et al. have reported down-regulation of miR-21 in the PBMCs of patients with insignificant coronary artery stenosis compared with CAD patients or healthy subjects. Levels of this miRNA have been negatively correlated with the PTEN. Moreover, they reported a gradual elevation miR-25 expression from healthy subjects to those with insignificant coronary artery stenosis and CAD patients. Expression levels of miR-21 and miR-25 in the PBMCs could differentiate three groups of study participants (39). Yao et al. have demonstrated the capacity of miRNAs in distinguishing CAD patients with heart failure (HF) from those without HF. Among the most significantly dysregulated miRNAs between these two groups of patients have miR-221, miR-19b-5p, and miR-25-5p. Combination of expression levels of these miRNAs in PBMCs and hypertension have been significantly correlated with higher risk of HF risk in CAD patients (40). Another miRNA with promising results in diagnostic approaches is miR-122-5p which could differentiate unstable CAD patients from healthy controls with accuracy of 0.9, yet its accuracy in differentiation of stable patients from controls was not appropriate (41). A brief review of studies which demonstrated this function is presented in Table 4.

**Table 4:** Diagnostic/prognostic significance of miRNAs in CAD (UA, unstable angina; STEMI: ST-segment elevation myocardial infarction).

**microRNA**	**Expression pattern**	**Samples**	**Diagnostic/prognostic role**	**ROC curve analysis**			**References**
				**Sensitivity**	**Specificity**	**AUC**	
*miR-142-3p*	Upregulated	Blood samples from 52 CAD patients and 26 normal subjects	Distinguishing UA patients from normal subjects	–	–	0.805	(38)
*miR-142-3p*	Upregulated	Blood samples from 52 CAD patients (including 26 patients with UA and 26 patients with STEMI) and 26 normal subjects	Distinguishing STEMI patients from normal subjects	–	–	0.840	
*miR-17-5p*	Upregulated	Blood samples from 52 CAD patients (including 26 patients with UA and 26 patients with STEMI) and 26 normal subjects	Distinguishing STEMI patients from normal subjects	–	–	0.845	
*miR-223*	Upregulated	Plasma samples from 300 patients with coronary heart disease and 100 controls	Diagnostic biomarker	0.86	0.913	0.933	(42)
*miR-223-3 p*	Upregulated	Serum samples from 314 patients with unstable CAD, 389 patients with stable CAD and 442 controls	Discriminating unstable CAD patients from controls	–	–	0.76	(41)
*miR-122-5 p*	Upregulated	Serum samples from 314 patients with unstable CAD, 389 patients with stable CAD and 442 controls	Discriminating unstable CAD patients from controls	–	–	0.90	
*miR-223-3 p* *miR-122-5 p* along with age and gender	Upregulated Upregulated	Serum samples from 314 patients with unstable CAD, 389 patients with stable CAD and 442 controls	discriminating unstable CAD patients from controls	–	–	0.96	
*miR-122-5 p*	Upregulated	Serum samples from 314 patients with unstable CAD, 389 patients with stable CAD and 442 controls	discriminating stable CAD patients from controls	–	–	0.63	
*miR-223-3 p* *miR-122-5 p* along with age and gender	Upregulated Upregulated	Serum samples from 314 patients with unstable CAD, 389 patients with stable CAD and 442 controls	Diagnostic biomarker (discriminating stable CAD patients from controls)	–	–	0.80	
*miR-495-3p*	Upregulated	Blood samples (PBMCs) from 114 patients with stable CAD(including patients with prethrombotic status (PTS) and patients without PTS) and 24 healthy volunteers as controls	Discriminating PTS patients from non-PTS patients	–	–	0.712	(43)
*miR-34a-5p*	Upregulated	Blood samples (PBMCs) from 114 patients with stable CAD(including patients with prethrombotic status (PTS) and patients without PTS) and 24 healthy volunteers as controls	Discriminating PTS patients from non-PTS patients	–	–	0.780	
*miR-34a-5p*along with fibrinogen	Upregulated	Blood samples (PBMCs) from 114 patients with stable CAD(including patients with prethrombotic status (PTS) and patients without PTS) and 24 healthy volunteers as controls	Discriminating PTS patients from non-PTS patients	–	–	0.885	
*miR-93-5p*along with FHS risk factors	Upregulated	Plasma samples from 50 patients with stable CAD, 50 patients with STEMI and 50 controls	Distinguishing CAD patients from controls	–	–	0.77	(44)
*miR-499a-5p*along with FHS risk factors	Upregulated	Plasma samples from 50 patients with stable CAD, 50 patients with STEMI, and 50 controls	Distinguishing STEMI patients from controls	–	–	0.93	
*miR-146a*	Upregulated	Plasma samples from 34 CAD patients with good coronary collateral circulation (CCC) and 44 CAD patients with poor CCC	Discriminating CAD patients with good and poor CCC	–	–	0.939	(45)
*miR-208a*	Upregulated	Plasma samples from 290 patients with coronary heart disease (CHD) and 110 individuals without CHD	Diagnostic biomarker	0.75	0.93	0.919	(46)
*miR-208a*	Upregulated	Plasma samples from 95 patients with CAD and 50 individual without CAD	Diagnostic biomarker	–	–	0.819	(45)
*miR-370*	Upregulated	Plasma samples from 95 patients with CAD and 50 individual without CAD	Diagnostic biomarker	–	–	0.745	
*miR-208a* *miR-370*	Upregulated Upregulated	Plasma samples from 95 patients with CAD and 50 individual without CAD	Diagnostic biomarker	–	–	0.856	
*miR-21*	Upregulated	Serum samples from 45 patients with diabetes mellitus (DM) and CAD, 45 patients with DM and heart failure (HF), 45 patients with DM, and 45 matched control subjects	discriminating CAD + DM group from controls	0.800	0.911	0.944	(47)
*miR-21*	Upregulated	Serum samples from 45 patients with diabetes mellitus (DM) and CAD, 45 patients with DM and heart failure (HF), 45 patients with DM, and 45 matched control subjects	discriminating CAD + DM group from DM group	0.778	0.667	0.755	
*miR-21*	Upregulated	Serum samples from 45 patients with diabetes mellitus (DM) and CAD, 45 patients with DM and heart failure (HF), 45 patients with DM, and 45 matched control subjects	discriminating CAD + DM form HF + DM group	0.711	0.511	0.640	
*miR-21*	Upregulated (in ACS patients compared with CAD patients)	50 patients with acute coronary syndrome (ACS) and 50 patients with stable CAD	Distinguishing ACS patients from CAD patients	–	–	0.775	(48)
*miR-151-3p*	Upregulated (in STEMI group)	Plasma samples from 20 patients with STEMI, 20 patients with stable CAD and 20 individuals without CAD	Distinguishing patients with STEMI form non-CAD individuals	–	–	0.758	(49)
*miR-151-3p*	Upregulated (in STEMI group)	Plasma samples from 20 patients with STEMI, 20 patients with stable CAD and 20 individuals without CAD	Distinguishing patients with STEMI form patients with stable CAD	–	–	0.754	
*miR-331*	Upregulated (in STEMI group)	Plasma samples from 20 patients with STEMI, 20 patients with stable CAD and 20 individuals without CAD	Distinguishing patients with STEMI form non-CAD individuals	–	–	0.790	
*miR-331*	Upregulated (in STEMI group)	Plasma samples from 20 patients with STEMI, 20 patients with stable CAD and 20 individuals without CAD	Distinguishing patients with STEMI form patients with stable CAD	–	–	0.773	
*miR-221* *miR-25-5p* *miR-19b-5p*	Upregulated Upregulated Downregulated	50 CAD patients with heart failure and 48 CAD patients without heart failure	CAD patients with heart failure and CAD patients without heart failure	–	–	0.860	(40)
*miR-221* *miR-25-5p* *miR-19b-5p* together with hypertension	Upregulated Upregulated Downregulated	50 CAD patients with heart failure and 48 CAD patients without heart failure	CAD patients with heart failure and CAD patients without heart failure	–	–	0.871	
*miR-941*	Upregulated	Blood samples from 56 CAD patients [18 patients with STEMI, 18 patients non-ST elevation ACS (NSTE-ACS), and 20 patients with stable angina (SA)] and 16 patients without CAD	Distinguishing STEMI patients form patients without CAD	–	–	0.896	(50)
*miR-941*	Upregulated	Blood samples from 56 CAD patients (18 patients with STEMI, 18 patients non-ST elevation ACS (NSTE-ACS) and 20 patients with stable angina (SA)) and 16 patients without CAD	distinguishing STEMI patients form patients with SA	–	–	0.808	
*miR-941*	Upregulated	Blood samples from 56 CAD patients [18 patients with STEMI, 18 patients non-ST elevation ACS (NSTE-ACS), and 20 patients with stable angina (SA)] and 16 patients without CAD	Distinguishing STEMI patients form patients with NSTE-ACS	–	–	0.781	
*miR-133a*	Upregulated (in patients with PMI)	Serum samples from 80 CAD patients (48 patients with periprocedural myocardial injury (PMI) after percutaneous coronary intervention (PCI) and 32 patients without PMI)	Prognostic biomarker (predicting occurrence of PMI)	0.938	0.719	0.891	(51)
*miR-25*	Upregulated	Blood samples (PBMCs) from 72 CAD patients, 30 patients with ICAD and 74 controls	Distinguishing CAD patients from controls)	0.85	0.78	0.83	(39)
*miR-25*	Upregulated		Distinguishing CAD patients from patients with ICAD	0.57	0.76	0.66	
*miR-25*	Upregulated		Distinguishing ICAD patients from controls	0.62	0.88	0.76	
*miR-25*	Upregulated		Distinguishing CAD patients from other subjects	0.85	0.67	0.78	
*miR-21*	Downregulated (in ICAD group)		Distinguishing CAD patients from patients with ICAD	0.58	0.83	0.66	
*miR-21*	Downregulated (in ICAD group)		Distinguishing ICAD patients from controls	0.79	0.68	0.76	
*miR-218*	Downregulated	Serum samples from 104 CAD patients and 101 healthy controls	Diagnostic biomarker	0.86	0.86	0.889	(26)
*Let-7f* *miR-19a* *miR-126* *miR-210* *miR-296*	Downregulated Downregulated Downregulated Downregulated Downregulated	Plasma samples from 286 patients with CAD (including 113 patients with rapid angiographic stenotic progression (RASP) and 173 patients without RASP)	Distinguishing RASP patients from non-RASP patients	–	–	0.879	(51)
*miR-126*	–	Plasma samples from 46 patients with diabetes and CAD, 54 patients with diabetes but without CAD and 20 healthy controls	Discriminating diabetic patients with and without CAD	0.91	1	–	(52)
*miR-210*	–	Plasma samples from 46 patients with diabetes and CAD, 54 patients with diabetes but without CAD and 20 healthy controls	Discriminating diabetic patients with and without CAD	0.93	1	–	
*miR-378*	Downregulated	Plasma samples from 215 CAD patients and 52 matched healthy subjects	Diagnostic biomarker	–	–	0.789	(53)
*let-7c*	Downregulated	Plasma samples from 69 CAD patients and 30 control individuals	Diagnostic biomarker	–	–	0.654	(54)
*miR-145*	Downregulated		Diagnostic biomarker	–	–	0.670	
*miR-155*	Downregulated		Diagnostic biomarker	–	–	0.620	
*let-7c* *miR-145* *miR-155*	Downregulated Downregulated Downregulated		Diagnostic biomarker	–	–	0.706	
*miR-132*	–	Serum samples from 1112 patients with CAD (682 patients with stable angina pectoris and 430 patients with acute coronary syndrome)	Prognostic biomarker (prediction of cardiovascular death)	–	–	0.737	(55)
*miR-140-3p*	–		Prognostic biomarker (prediction of cardiovascular death)	–	–	0.756	
*miR-210*	–		Prognostic biomarker (prediction of cardiovascular death)	–	–	0.754	
*miR-150*	–	Blood samples (PBMCs) from 72 CAD patients with significant stenosis, 30 CAD patients with insignificant stenosis (ICAD) and 74 healthy controls	discriminating CAD patients from healthy controls)	0.90	0.62	0.79	(56)
*miR-223*	–		discriminating CAD patients from healthy controls)	0.37	0.91	0.62	
*miR-150* *miR-223*	–		Discriminating CAD patients from healthy controls)	0.89	0.65	0.79	
*miR-150*	-		Discriminating CAD patients form ICAD patients	0.40	0.96	0.70	
*miR-223*	–		Discriminating CAD patients form ICAD patients	0.55	0.89	0.71	
*miR-150miR-223*	–		Discriminating CAD patients form ICAD patients	0.74	0.83	0.80	
*miR-423-3p*	–	Serum samples form 64 CAD patients and 2,748 control individuals	Diagnostic biomarker	–	–	0.8	(57)
*miR-26*	Downregulated	45 patients with type 2 diabetes, 45 patients with type 2 diabetes and CAD and 45 healthy controls	Discriminating patients with type 2 diabetes and CAD from healthy controls	–	–	0.948	(58)
*miR-26*	Downregulated	45 patients with type 2 diabetes, 45 patients with type 2 diabetes and CAD and 45 healthy controls	discriminating type 2 diabetes patients with and without CAD	–	–	0.807	
*miR-196-5p*	Downregulated	60 patients with early-onset CAD and 60 age- and gender-matched normal subjects	Diagnostic biomarker	0.85	0.72	0.824	(59)
*miR-3163-3p*	Downregulated	60 patients with early-onset CAD and 60 age- and gender-matched normal subjects	Diagnostic biomarker	0.57	0.84	0.758	
*miR-145-3p*	Downregulated	60 patients with early-onset CAD and 60 age- and gender-matched normal subjects	Diagnostic biomarker	0.67	0.82	0.753	
*miR-190a-5p*	Downregulated	60 patients with early-onset CAD and 60 age- and gender-matched normal subjects	Diagnostic biomarker	0.70	0.75	0.782	
*miR-196a*	Downregulated	72 patients with CAD, 30 patients with ICAD and 74 healthy controls	distinguishing ICAD patients from CAD patients	–	–	0.75	(60)

## miRNA Polymorphisms and Copy Number Variations in CAD

Both single nucleotide polymorphisms (SNPs) and copy number variations (CNVs) within miRNA coding genes have been associated with risk of CAD. Sung et al. have examined the relation between miR-146a, miR-149, miR-196a2, and miR-499 SNPs and CAD in a Korean population. They have reported association between the miR-149 rs2292832 and miR-196a2 rs11614913 SNPs and this disorder. Notably, the miR-146a rs2910164 GG genotype has been more prevalent among CAD patients with more than two stents. Moreover, combination of miR-146a G, miR-149 T, miR-196a2 C, and mIR-499 G alleles has been considerably associated with CAD occurrence. Certain SNPs have been reported to increase susceptibility to CAD in different subclasses of study participants such as non-smokers, hypertensive and non-diabetic individuals (61). Sohrabifar et al. have evaluated the presence of CNVs of hsa-miR-93, hsa-miR-122, hsa-miR-192 in CAD patients with or without type 2 diabetes mellitus. They have reported remarkable differences in the distribution of CNVs of hsa-miR-93 between CAD and non-CAD as well as between diabetic CAD and diabetic non-CAD individuals. In addition, hsa-miR-122 CNVs have been differently distributed among three subgroups (62). The rs2292832 miR-149 has been associated with risk of CAD in Iranian population. However, this SNP does not either affect the secondary structure of pre-miR-149 or the stability of the miRNA hairpin structure (63). As this SNP is located outside the sequence of mature miR-149, it has been proposed that it might affect the maturation process and therefore decrease expression of miR-149 (64). T allele of rs2431697 in miR-146a has been associated with higher risk of CAD (65). In addition, the rs2910164 within this miRNA affects risk of CAD (61). This SNP resides in the precursor of miR-146a and results in down-regulation of levels of mature miR-146a (66). Table 5 reviews the investigations which appraised the role of SNPs/CNVs in conferring risk of CAD.

**Table 5:** miRNA polymorphisms in CAD.

**microRNA**	**Polymorphism**	**Samples**	**Population**	**Assay method**	**Association**	**References**
*miR-196a2*	SNP (rs11614913)	Blood samples from 505 CAD patients and 1,109 control subjects	Chinese	SNPscan™ genotyping assay	Was associated with reduced risk of myocardial infarction and also was correlated with reduced risk of CAD in females	(67)
*miR-196a2*	SNP (rs11614913)	Blood samples form 218 CAD patients and 611 healthy individuals	Mexican	5′ exonuclease TaqMan assays	T allele of this polymorphism was correlated with elevated risk of CAD	(68)
*miR-196a2*	SNP (rs11614913)		Greek population	PCR-RFLP, High resolution Melting (HRM), and Sanger sequencing	This polymorphism was correlated with elevated risk of CAD	(69)
*miR-499*	SNP (rs3746444)	Blood samples form 200 CAD patients and 200 healthy individuals as controls	Greek population		This polymorphism was correlated with elevated risk of CAD	
*miR-196a2*	SNP (rs11614913)	Blood samples from 522 CAD patients and 535 control individuals	South Korean	PCR-RFLP	Is associated with enhanced risk of CAD in females and patients aged >63 years old. Also correlated with prevalence of CAD	(61)
*miR-149*	SNP (rs2292832)		South Korean	PCR-RFLP	Is associated with enhanced risk of CAD in females and patients aged >63 years old. Also correlated with prevalence of CAD	
*miR-146a*	SNP (rs2910164)		South Korean	PCR-RFLP	GG genotype of this SNP was correlated with risk of CAD in stent ≥2 group. Also this polymorphism was associated with elevated risk of CAD in non-smoking, hypertensive and non-diabetic subgroups	
*miR-146a*	SNP (rs2431697, rs2910164)	Blood sample from 353 patients with CAD and 368 control subjects	Chinese	Sequenom MassARRAY system and matrix-assisted laser desorption/ionization time-of-flight mass spectrometry	Carriers of T allele in rs2431697 had enhanced risk of CAD. G allele of rs2910164 was associated with reduced risk of CAD.	(65)
*miR-423*	SNP (rs6505162)	Blood samples from 100 patients with CAD and 117 gender-matched healthy subjects	Indian	ARMS-PCR	A allele and CA genotype of this SNP was associated with augmented risk of CAD	(70)
*miR-224*	SNP (rs188519172)	Blood samples from 100 CAD patients and 100 matched healthy subjects	–	ARMS-PCR	GA genotype of this SNP was associated with reduced CAD predisposition	(71)
*miR-4513*	SNP (rs2168518)	100 CAD patients and 100 healthy controls	Indian	ARMS-PCR	T allele and CT genotype of this SNP was correlated with enhanced predisposition to CAD	(71)
*pre-mir-499*	SNP (rs3746444)	288 patients with CAD and 150 control subjects	Iranian	PCR-RFLP	Frequency of GG genotype of this SNP was significantly higher in CAD patients than controls	(63)
*miR-149*	SNP (rs2292832)	272 patients with CAD and 149 control subjects	Iranian	PCR-RFLP	TT genotype of rs2292832 was associated with CAD risk	(63)
*hsa-miR-93*	copy number variation (CNV)	Blood samples from 50 CAD patients (25 diabetic and 25 non-diabetic) and 50 subjects without CAD (25 diabetic and 25 non-diabetic)	Iranian	Real-time PCR	CNVs in hsa-miR-93 were significantly different between CAD patients and non-CAD subjects. CNVs of this miRNA were significantly different between CAD patients CAD patients type 2 diabetes mellitus (T2DM) and non-CAD individuals without T2DM.	(62)
*hsa-miR-192*	CNV		Iranian	Real-time PCR	CNVs of hsa-miR-192 were significantly different between CAD patients with T2DM and non-CAD individuals without T2DM.	
*hsa-miR-122*	CNV		Iranian	Real-time PCR	CNVs of hsa-miR-122 were significantly different between: CAD patients and non-CAD subjects CAD patients with T2DM and CAD patients without T2DM CAD patients with T2DM and non-CAD individuals without T2DM	

## Conclusions and Perspectives

Aberrant expression of miRNAs in CAD patients has been recognized through high throughput sequencing methods in addition to candidate gene assays. An example of the former type of assays has been conducted through investigation of Gene Expression Omnibus (GEO) database showing frequent differential expression of 150 genes and 5 miRNAs (22). Luciferase reporter assays have shown the functional interactions between a number of miRNAs and mRNAs (24, 72). miRNAs can regulate development of CAD through different mechanisms such as modulation of angiogenesis [miR-92a-3p (13), miR-939 (20), and miR-206 (14)], inflammatory responses [miR-181a-5p, miR-181a-3p (21), miR-216a (16), and miR-383-3p (19)], leukocyte adhesion [miR-21 (37) and miR-25 (39)] and modulation of activity of VSMCs [miR-574-5p (27)]. Notably, a number of miRNAs influence different aspects of this process or different targets in a certain process. For instance, miR-206 regulated expressions of VEGF, PIK3C2α, Akt, and endothelial nitric oxide synthase, all of them being involved in the angiogenic processes. NF-Kβ/TNF-α, PI3K-Akt-mTOR, WNT, and VEGFA/ERK1/2/NF-κB are among signaling pathways which are regulated by miRNAs in the context of CAD.

In addition to dysregulation of expression of miRNAs in endothelial cells and VSMCs, microvesicles originated from these cells have been shown to contain abnormal levels of miRNAs, thus these particles can broaden the extent of miRNAs effects on diverse cells. The presence of miRNAs in the circulation of CAD patients endowed them the ability to predict disease course and distinguish CAD patients from healthy subjects. Both plasma and PBMC levels of miRNAs could be used as diagnostic markers for CAD. Most importantly, miRNAs signature can predict the occurrence of CAD-related complications such as HF. Their ability in distinguishing UA from MI is another promising result of recent investigations, potentiating them as accurate diagnostic marker for stratifying patients who need urgent interventions. However, a major limitation of application of miRNAs as diagnostic or prognostic markers in CAD is the influence of other age-related factors on their expression. Identification of CAD-specific miRNAs whose expressions are not affected by patients' health condition is a major issue in this regard. Longitudinal assessment of miRNA profile in relation with health status of CAD patients and measurement of possible confounding parameters would help in identification of markers for clinical application.

Finally, several SNPs and CNVs within miRNA coding genes have been associated with risk of CAD, providing further evidence for crucial partake of miRNAs in the pathogenesis of CAD. Most notably, some genotypes of these SNPs have been associated with risk of CAD in patients with specific lifestyles or habits (61, 62), demonstrating the possible interaction between these genetic variants and environmental factors. However, the impact of these SNPs on CAD-related biological processes such as cell adhesion, inflammation, proliferation or apoptosis has not been appraised *in vitro*. Conduction of these types of studies would pave the way for design of targeted therapeutic interventions in CAD. Taken together, miRNAs participate in different aspects of CAD pathogenesis and could be used as specific/sensitive markers for this condition. The therapeutic application of miRNAs in CAD should be judged in upcoming studies.

## Author Contributions

MT and SG-F wrote the draft and revised it. MG designed the tables and collected the data. All authors contributed to the article and approved the submitted version.

## Conflict of Interest

The authors declare that the research was conducted in the absence of any commercial or financial relationships that could be construed as a potential conflict of interest.

## Abbreviations

ARMS: Amplification-Refractory Mutation System
ANGII: angiotensinogen
TAB2, binding protein 2, CNVs: Copy Number Variations
CCL2: C-C motif chemokine ligand 2
copy number variations
CAD: Coronary artery disease
EPCs: endothelial progenitor cells
ET-1, endothelin 1;GEO: Gene Expression Omnibus
HF: heart failure
HRM: High resolution Melting
HUVECs: human umbilical vein endothelial cells
ICAM1: intercellular adhesion molecule 1
KRT1: keratin 1
LDL: low-density lipoprotein
LPS: lipopolysaccharide
miRNAs: MicroRNAs
MI: myocardial infarction
MVs: microvesicles
MAPK8: mitogen-activated protein kinase 8
MEF2C: myocyte enhancer factor 2C
MEF2C: myocyte enhancer factor 2C
IκBα: NF-κB inhibitor alpha
NRIP1: nuclear receptor interacting protein 1
eNOS: nitric oxide synthase 3
PBMCs: peripheral blood mononuclear cells
PIK3C2α: phosphatidylinositol-4-phosphate 3-kinase catalytic subunit type 2 alpha
PDCD4: programmed cell death 4
RFLP: Restriction fragment length polymorphism
STEMI: ST-segment elevation myocardial infarction
SNPs: single nucleotide polymorphisms
SMAD3: SMAD family member 3
SPF: specific-pathogen-free
THBS1: thrombospondin 1
MAP3K7: TGF-beta activated kinase 1
TxA2: thromboxane A2 TxA2
TRF2: telomeric repeat binding factor 2
TRAF6: TNF receptor associated factor 6
UA: unstable angina
VEGF: vascular endothelial growth factor
VSMCs: vascular smooth muscle cells
VCAM1: vascular cell adhesion molecule 1
ZDHHC14: zinc finger DHHC-type palmitoyltransferase 14.
